# Psychometric properties of the Romanian version of the female sexual function index (FSFI-RO)

**DOI:** 10.1186/s12905-023-02676-7

**Published:** 2023-10-06

**Authors:** Ana-Maria Cristina Daescu, Liana Dehelean, Dan-Bogdan Navolan, Gheorghe Nicusor Pop, Dana Liana Stoian

**Affiliations:** 1 PhD School Department, Victor Babes University of Medicine and Pharmacy, Timisoara, 300041 Romania; 2 Neurosciences Department, Victor Babes University of Medicine and Pharmacy, Timisoara, 300041 Romania; 3 Department of Obstetrics and Gynecology, Victor Babes University of Medicine and Pharmacy, Timisoara, 300041 Romania; 4 Department of Cardiology, Victor Babes University of Medicine and Pharmacy, Timisoara, 300041 Romania; 5 Department of Internal Medicine, Victor Babes University of Medicine and Pharmacy, Timisoara, 300041 Romania

**Keywords:** FSFI, Romanian version, FSFI-RO, Psychometric validation, Female sexual dysfunction

## Abstract

**Background:**

Female sexual dysfunction (FSD) is a highly prevalent health disorder and no self-report questionnaire on female sexual function is available in Romanian. Therefore we considered the Female Sexual Function Index (FSFI) to be the most appropriate due to its excellent psychometric properties. The FSFI is a measuring scale with 19 items that assess the six domains of female sexual function: desire, arousal, lubrication, orgasm, satisfaction and pain. The paper aims to analyze the psychometric reliability and validity of the FSFI-RO (Romanian Version of the Female Sexual Function Index).

**Methods:**

385 women (aged 18 to 51) enrolled in the present study. To assess the presence of FSD we used the Diagnostic and Statistical Manual of Mental Disorders, 5th edition (DSM-5) criteria for sexual dysfunction. Then we categorized the participants into two groups: the FSD group (41%) and the healthy control group (59%). Women were then asked to fill out a form that included sociodemographic information and the FSFI-RO questionnaire. A sample of 50 women agreed to re-answer FSFI-RO in a 4-week interval in order to evaluate the test-retest validity of the questionnaire. The data were summarized using descriptive statistics: the test-retest reliability was measured by the intraclass correlation coefficient (ICC); Cronbach’s alpha was employed to evaluate the internal consistency of the Romanian version of the FSFI, and validity was assessed by the content and construct validity.

**Results:**

The results showed high test-retest reliability, with ICC from 0.942 to 0.991 in the domains and 0.987 in the total score. Regarding the internal consistency of the FSFI-RO, Cronbach’s α coefficients were found to be high (α = 0.944). Convergent construct validity proved to be moderate to high in desire, arousal, lubrication, orgasm and, satisfaction domains, and weak correlation in the pain domain. Regarding the discriminant construct validity, the scores for each domain and the total score showed statistically significant differences between the FSD group and the control group.

**Conclusions:**

The FSFI-RO showed similar psychometric properties to those of the original version, therefore being a reliable and valid instrument that can be used in Romanian-speaking women.

## Background

Sexual function is the consequence of a complex neurovascular process influenced by biological (age, levels of sex hormones, gynecological/obstetrical history, chronic medical conditions), psychological (mental health, personality traits, life satisfaction, self-esteem, attitude towards sexuality), social (religion, social support) and interpersonal factors (relationship quality, availability of a partner) [1, 2]. Also, external factors, such as environmental stimuli, including erotic cues, social context, and interpersonal dynamics, can significantly influence and shape sexual response. The domains of sexual function, as defined by the Sexual Function Index (FSFI) are: desire, arousal, lubrication, orgasm, satisfaction, and pain. The desire domain assesses a woman’s level of sexual interest or libido, including the frequency and intensity of sexual thoughts, fantasies, and desires. The arousal domain focuses on a woman’s physiological and subjective sexual arousal response. It encompasses factors such as the presence and intensity of sexual excitement, genital sensations, lubrication, and overall sexual responsiveness. The lubrication domain within the FSFI evaluates the adequacy of vaginal lubrication during sexual activity. The orgasm domain measures a woman’s ability to reach climax or experience sexual release. It considers factors such as the frequency, intensity, and satisfaction associated with orgasms. The satisfaction domain assesses overall sexual satisfaction and fulfillment. It encompasses feelings of contentment, pleasure, emotional closeness, and general satisfaction with one’s sexual relationship. The pain domain focuses on any pain or discomfort experienced during sexual activity, including factors such as pain during penetration or persistent pain before, during, or after sexual intercourse [1, 4]. Female sexual dysfunction (FSD) is a highly prevalent health disorder, as it affects 41% of premenopausal women around the globe, making it a significant public health problem and raising concern regarding female sexual function [3]. FSD is described as “the various ways in which a woman is unable to participate in a sexual relationship as she would wish” [4]. A clinical approach to the definition of FSD states that it is “the persistent/recurring decrease in sexual desire or arousal, the difficulty/inability to achieve an orgasm, and/or the feeling of pain during sexual intercourse” [5]. The Diagnostic and Statistical Manual of Mental Disorders, 5th edition (DSM-5) criteria for female sexual dysfunction require that the sexual symptoms must be present for a minimum duration of approximately 6 months, causing clinically significant distress in the individual. Also, nonsexual mental disorders, severe relationship distress, or other significant stressors must not be present. Sexual symptoms are not attributable to the effect of medication or another medical condition [6].

Significant risk factors for female sexual dysfunction were identified in recent studies: poor physical and mental health, stress, abortion, genitourinary issues, female genital mutilation, relationship dissatisfaction, sexual abuse, and religious beliefs. The study also included significant protective factors, like: exercise, older age at marriage, daily affection, intimate communication, having a healthy body image, and sex education [1].

In psychiatry, rating scales are at the base of any quantitative research study and are frequently regarded as the equivalent of any other medical report or investigation. Rating scales aid in the classification and quantification of the subject to be studied [7]. Various screening and diagnostic tools regarding female sexual function have been validated for use in women, some being thoroughly tested for psychometric reliability: The Derogatis Interview for Sexual Function [8], The Brief Index of Sexual Functioning in Women [9], The Female Sexual Function Index (FSFI) [10] along with its short form, FSFI-6 [11], The Changes in Sexual Function Questionnaire [12], Fallowfield’s Sexual Activity Questionnaire (FSAQ) [13]. These measures have been validated in various international populations, but they are not available for Romanian-speaking women.

Although widely criticized (the FSFI was originally designed to measure specifically arousal disorders; this aspect calls into question the measurement’s validity to the other domains of sexual function; it was hypothesized that the FSFI would not give and adequate measurement of other sexual issues, besides sexual arousal disorders), the FSFI remains one of the most widely used questionnaires to evaluate sexual health in female population [14]. It is a quantifiable comparison standard recognized and used by many researchers in studies regarding female sexual function [15–17]. There are many alternatives to the FSFI and all of them have good psychometric qualities in terms of reliability and validity, but the fact that many of them have not been validated across different cultures and have not been translated into multiple languages is a significant restriction. Moreover, some of them have only been examined once during the development phase and they have not been used extensively in research. Review studies conducted in 2019 showed that FSFI is a suitable screening tool for FSD, showing strong evidence for criterion validity [18].

The FSFI was developed and validated in the year 2000 by a group of researchers studying the female sexuality field and it has been regarded as the gold standard for measuring female sexual function for the past 20 years [19]. The FSFI is a self-report, 19-item test of female sexual function that gives results on both the general level of sexual function and the primary elements of female sexual function, such as sexual desire, arousal, orgasm, pain, and satisfaction [19]. The FSFI desire domain addresses the woman’s motivation for sexual activity in terms of level and frequency of having sexual desire; the FSFI arousal domain includes items that assess arousal frequency and intensity, and also confidence in one’s abilities to become aroused; the items in the lubrication domain evaluate the frequency and difficulty of attaining and maintaining lubrication during sexual activity; the orgasm domain evaluate a woman’s ability to reach orgasm; the satisfaction domain addresses partner satisfaction and satisfaction with overall sexual function; and the pain domain’s items evaluate pain during or after sexual activity [19].

In the initial psychometric validation phase, the FSFI showed excellent internal reliability (Cronbach’s alphas > 0.9 for all subscales) and good test-retest reliability (r = 0.79–0.88). Construct validity was demonstrated by highly significant mean difference scores between the sexually functional and dysfunctional groups for each of the domains (p ± 0.001) [10].

The FSFI is a widely used screening instrument for the presence of any type FSD. The FSFI was cross-validated in women with a wide variety of sexual disorders [20]. The questionnaire has been translated and validated in other languages like Italian [21], Spanish [22], French [23], Greek [24], Portuguese [25], Hungarian [26], Polish [27], Chinese [28], Arabic [29], Persian [30], Malay [31], Japanese [32], Iranian [33], Vietnamese [34] and Urdu [35]. It has been used to evaluate sexual symptoms in women with various clinical conditions, like: infertility [36], vaginismus [37], pelvic floor disorders [38], female genital mutilation [39], pregnancy [40], polycystic ovary syndrome [41], thyroid autoimmune disease [47], diabetes mellitus [42], hypertension [43], obesity [44], metabolic syndrome [45], and COVID-19 [46].

The Romanian translation of the FSFI has been used in studies conducted in Romania [47, 48], but a study on the psychometric properties of the Romanian version does not exist.

Previous research has found that women are often hesitant to seek care for sexual health difficulties [49]. Sexual dysfunction can lead to a significant decline in quality of life in both men and women [2]. FSD is more complex and significantly less studied and understood in comparison with male sexual dysfunction [50]. In Romania, even a smaller number of women address their doctors regarding sexual conditions, probably due to economic, socio-cultural, and religious considerations. No scientific study has been conducted in Romania, regarding the prevalence of FSD, but general physicians, gynecologists, endocrinologists, urologists, andrologists, and psychiatrists estimate that the numbers are similar to those reported in other parts of the world. No self-report questionnaire on female sexual function or other screening/diagnostic instrument for FSD is available in the Romanian language, even though it is much needed. Therefore, this paper aims to contribute to the enrichment of knowledge in the field of female sexual function, by providing a validated Romanian version of the Female Sexual Function Index (FSFI) that could be used in clinical settings or in research on Romanian speaking population. We considered FSFI to be the most appropriate due to its excellent psychometric properties and high cultural adaptability. The paper aims to translate the FSFI into Romanian language and to measure its psychometric reliability and validity.

Specifically, we will assess internal consistency by calculating Cronbach’s alpha coefficient for each subscale of the Romanian FSFI, which will reflect the interrelatedness of items within each subscale. Furthermore, we will establish content validity through a rigorous translation and adaptation process, ensuring conceptual equivalence to the original questionnaire. Construct validity will be evaluated through factor analysis, comparing the factor structure of the Romanian FSFI with the original questionnaire. Additionally, we will examine convergent validity by exploring correlations between the Romanian FSFI subscales and other measures of sexual functioning and quality of life. By incorporating these assessments, we aim to provide robust evidence for the reliability and validity of the Romanian FSFI.

## Methods

### Translation of FSFI into Romanian

The original version of the FSFI was translated into Romanian by a physician (one of the authors) who is fluent in both English and Romanian. Then, to acquire a reliable translation, the FSFI-RO was reverse translated by an independent translator. After that, a member of the research team, who is an expert in the field of sexual medicine, compared the reverse translated version with the original version. The FSFI-RO was further reviewed by a four-member committee: a psychiatrist, an endocrinologist, and two gynecologists. The final refined and approved version was given to 50 women who entered a pilot study to test the questionnaire’s comprehensibility. The 50 women were then asked to participate in an interview to see if they had any difficulties understating and interpreting the items. No major issues were observed.

### Participants and procedure

Three hundred eighty-five women, aged between 18 and 51 (mean age 29.41 ± 6.39), enrolled in the present study. Women were selected from the SCJUPBT Outpatient Endocrinology Clinic in Timisoara, Romania. The written informed consent was obtained from all participants. Women above 18 years of age, who had been sexually active for at least 4 weeks before the study, having the ability to read and understand the Romanian language, were included in the study. The exclusion criteria were: pregnant women and up to 6 months postpartum, women with neurologic and psychiatric disorders, severe somatic disorders, women known with psychoactive substances abuse or dependence, illiterate women, and those taking medications that could affect sexual functioning, as well as women reporting they haven’t had any sexual activity in the past 4 weeks.

Following a brief interview focused on the personal medical history, the participants were asked to respond to questions related to their sexual health. To assess the presence of FSD we used the DSM-5 criteria for sexual dysfunction: the sexual symptoms must be present for a minimum duration of approximately 6 months, causing clinically significant distress in the individual; nonsexual mental disorders, severe relationship distress, or other significant stressors must not be present and sexual symptoms are not attributable to the effect of medication or another medical condition [6]. The interviews were conducted in a private and confidential setting by trained healthcare professionals experienced in using the DSM-5 criteria for diagnosing female sexual dysfunction. These professionals followed standardized protocols to ensure consistency and accuracy in the assessment process.

The participants were then divided into two groups, the FSD group (41%) and the healthy control group (59%).

Women were then asked to fill in the socio-demographic form and the FSFI-RO. The response rate was 87%. A sample of 50 women (19 from the FSD group and 31 from the control group) agreed to re-answer FSFI-RO in a 4-week interval in order to evaluate the test-retest validity of the questionnaire. No treatment was administered during this time, ensuring the patient’s symptoms did not alter.

The study was conducted according to the guidelines of the Declaration of Helsinki, and approved by the Local Ethics Committee of SCJUPBT, Timisoara, Romania (225/12.02.2021).

### Instruments

The instruments used in this study were: a socio-demographic form (the data collected were: age, education, religion, marital status, area of residence, and occupational status); DSM-5 Criteria for Sexual Dysfunction, and the FSFI-RO.

#### DSM-5 criteria for sexual dysfunction

The DSM-5 provides diagnostic criteria for sexual dysfunction, being used internationally for the diagnostic and classification of sexual dysfunction both in males and females. [6]

#### FSFI

The FSFI comprises 19 items divided into six domains: desire, arousal, lubrication, orgasm, satisfaction, and pain. Desire and satisfaction items are rated on a 5-point Likert scale, ranging from 1 to 5, and the other items are rated on a 6-point Likert scale, ranging from 0 to 5. The full- scale score ranges between 2 and 36 points. The full scale score is calculated after summing all six domains scores. For individual domain scores, the total is calculated by adding the individual scores of the domain’s constituent items and multiply by the domain factor (the factor for each domain is presented in Table 1). It should be noted that a subject reporting no sexual activity in the previous month receives a domain score of zero for each individual domain. A threshold value of ≤ 26.55 was established for detecting FSD [20].

**Table 1:** Female Sexual Function Index (FSFI) Scoring Appendix

Domain	Score Range	Factor	Minimum Score	Maximum Score
Desire	1–5	0.6	2	30
Arousal	0–5	0.3		
Lubrication	0–5	0.3		
Orgasm	0–5	0.4		
Satisfaction	1–5	0.4		
Pain	0–5	0.4		
Full-Scale Score Range				

### Statistical analysis

The data were summarized using descriptive statistics. Numeric variables were presented as mean and standard deviation (SD) or median and quartiles [Q1-Q3], and categorical variables were presented as frequency and percentages. We employed the Shapiro-Wilk test in order to check for the Gaussian distribution of numeric variables. To compare the general study population characteristics, we employed the Mann-Whitney U test for numerical variables and the Chi-square test with Yates’ correction where applicable for nominal variables.

The test-retest reliability was measured by the intraclass correlation coefficient (ICC) for each domain and the total score. Values between 0.6 and 0.8 represent good agreement, while values > 0.8 represent excellent agreement for test-retest reliability [51].

Cronbach’s alpha was employed to evaluate the internal consistency of the Romanian version of the FSFI. A Cronbach’s alpha coefficient of 0.7 is considered acceptable, higher values correspond to a greater internal consistency [52]. Additionally, for the evaluation of internal consistency, we also used McDonald’s Omega coefficient. The 95% confidence interval was calculated by the Bootstrap method using 10’000 samples.

Validity was assessed by the content and construct validity (factorial, convergent, and discriminant). Factor structure was assessed with principal component analysis and subsequent confirmatory factor analysis. A Promax rotation with Kaiser normalization was used in the total sample (n = 385) at baseline to evaluate the principal components or factors. A value of Kaiser-Meyer-Olkin (KMO) > 0.80 was considered optimal, while a value < 0.50 was considered insufficient [29]. The Bartlett test of sphericity was calculated to ascertain the correlations between the variables and the appropriateness of the factor model. For the Confirmatory Factor Analysis (CFA), we used goodness of fit indices - Comparative Fit Index (CFI) and Tucker-Lewis Index (TLI) for internal validity. The Standardized Root Mean Square Residual (SRMR) was also calculated. The CFA was performed in R using lavaan package (v0.6, Rosseel Y. et all, 2022). Convergent construct validity was measured in all FSFI-RO domains and the total score by using the Spearman rank correlation. The discriminant validity was assessed by comparing the FSFI-RO domains and the total score in the two groups by employing the Mann-Whitney U test followed by effect size calculation using Fritz, Moris & Richler formula (r = z/sqrt(N)). A value of r < 0.3 was considerate as small effect, r = 0.3–0.5 medium effect, and r > 0.5 large effect.

Data analysis was performed using the IBM SPSS Statistics for Windows, Version 26.0 (Armonk, NY: IBM Corp, USA) and R version 4.2.1 (R Core Team (2021). R: A language and environment for statistical computing. R Foundation for Statistical Computing, Vienna, Austria. A p-value lower than 0.05 was considered statistically significant.

#### Sample size and power

For this study no prior sample size calculation were employed. The n = 385 represents the number of patients selected for the study from our clinic. A post-hoc statistical power analysis was employed using the t-test in R (v 4.2.1, R Core Team, Vienna, Austria) and from the pwr package [53]. The 385 patients included in the present study provides a 99.99% power to detect a medium effect size (r = 0.5) between FSFI-RO scores of the two groups (alpha = 0.05, two-sided).

Regarding validation study, the literature research showed that there are no general criteria for the required sample size in a validation study. A sample size of at least 50–100 participants is generally recommended [54].

## Results

### General

Participants in this study were asked to fill in a socio-demographic form. The response rate of the participants was 90.5%. The data collected were: age, education, religion, marital status, area of residence, and occupational status. The general study population characteristics are presented in Table 2. Based on the presence of FSD, assessed using the DSM-5 criteria, participants were divided into two groups: the FSD group and the control group. No significant differences were observed regarding population characteristics, meaning the two groups are homogenous.

**Table 2:** General characteristics of the participants (n = 385)

	FSD group (n = 158)	Control group (n = 227)	p
Age	29 [25–34.2]	28 [24–32]	0.550 ^a^
Area of residence: Rural Urban	26 (43.3%) 132 (40.6%)	34 (56.7%) 193 (59.4%)	0.694 ^b^
Education: College Junior college High school Primary school	115 (40.1%) 6 (42.9%) 27 (44.3%) 10 (43.5%)	172 (59.9%) 8 (57.1%) 34 (55.7%) 13 (56.5%)	0.930 ^b^
Marital status: Married In a stable relationship Single	88 (41.1%) 56 (39.4%) 14 (48.3%)	126 (58.9%) 86 (60.6%) 15 (51.7%)	0.677 ^b^
Religion: Agnostic Atheist Catholic Orthodox Protestant	0 0 16 (53.3%) 138 (41.6%) 4 (26.7%)	4 (100%) 4 (100%) 14 (46.7%) 194 (58.4%) 11 (73.3%)	0.301^b^
Occupation: Employee Housewife Unemployed Student	123 (40.5%) 11 (39.3%) 7 (36.8%) 17 (50%)	181 (59.5%) 17 (60.7%) 12 (63.2%) 17 (50%)	0.719 ^b^
Desire Arousal Lubrication Orgasm Satisfaction Pain Total FSFI-RO Score	3 [2.4–3.6] 3.3 [2.4–3.9] 4.2 [3.3–4.8] 2.8 [1.6–4] 3.6 [2.4–4.8] 4.4 [2.8–5.6] 21.9 [17.5–24.4]	4.2 [3.6–4.8] 5.4 [4.8–5.7] 5.7 [5.1–6] 5.2 [4.8–6] 5.2 [4.8–6] 6 [4.8–6] 30.8 [28.7–32.4]	< 0.001 ^a^ < 0.001 ^a^ < 0.001 ^a^ < 0.001 ^a^ < 0.001 ^a^ < 0.001 ^a^ < 0.001 ^a^

FSD: Female Sexual Dysfunction; FSFI-RO: Female Sexual Function Index Romanian Version;

^a^ – Mann-Whitney U test; ^b^ – Chi-square test;

Numeric variables were presented as mean and standard deviation (SD) or median and quartiles [Q1-Q3], and categorical variables were presented as frequency and percentages.

### Validity

Convergent construct validity, calculated with the domain intercorrelations and correlations between each domain and the total FSFI-RO score, using Pearson’s correlation coefficient, proved to be moderate to high in desire, arousal, lubrication, orgasm and satisfaction domains, and weak correlation in the pain domain. The values were higher in the correlation of the total FSFI-RO score and the domains than in interdomains (Table 3).

**Table 3:** FSFI-RO domain intercorrelations (n = 385)

FSD group	Desire	Arousal		Lubrication	Orgasm	Satisfaction	Pain
Desire	1						
Arousal	0.496*		1				
Lubrication	0.165*		0.469*	1			
Orgasm	0.084		0.438*	0.215*	1		
Satisfaction	0.308*		0.568*	0.356*	0.369*	1	
Pain	0.039		-0.029	0.298*	-0.099	-0.041	1
Total FSFI-RO	0.517*		0.761*	0.669*	0.570*	0.672*	0.333*
**Control group**							
Desire	1						
Arousal	0.373*		1				
Lubrication	0.115		0.258*	1			
Orgasm	0.007		0.411*	0.302*	1		
Satisfaction	0.263*		0.445*	0.171*	0.299*	1	
Pain	-0.011		0.152*	0.097	0.098	0.160*	1
Total FSFI-RO	0.526*		0.706*	0.456*	0.577*	0.661*	0.411*
**Total Group**							
Desire	1						
Arousal	0.573*		1				
Lubrication	0.350*		0.634*	1			
Orgasm	0.306*		0.717*	0.566*	1		
Satisfaction	0.481*		0.751*	0.544*	0.618*	1	
Pain	0.207*		0.396*	0.416*	0.307*	0.366*	1
Total FSFI-RO	0.622*		0.900	0.737*	0.800*	0.837*	0.566*

FSD: Female Sexual Dysfunction; FSFI-RO: Female Sexual Function Index Romanian Version; The values in the table represent Pearson’s correlation coefficient; * significance threshold reached (p < 0.05), with a confidence interval of 95%CI

To evaluate the discriminant construct validity between the FSD group and the control group, the domain scores and the total scores of both groups were compared. In Table 4 we present the scores for each FSFI-RO domain and the total FSFI-RO scores, which showed statistically significant differences between the FSD group and the control group in all domains and the total score. We found a medium effect size for desire and pain. For arousal, lubrication, orgasm, satisfaction, and total score, a large effect size was observed. This values indicates a practical significance of the results.

**Table 4:** Discriminant construct validity (n = 385)

	FSD group (n = 158)	Control group (n = 227)	Standardized test statistics z	Effect Size (r)	p*
Desire	3 [2.4–3.6]	4.2 [3.6–4.8]	-9.06	0.46	< 0.001
Arousal	3.3 [2.4–3.9]	5.4 [4.8–5.7]	-15.48	0.79	< 0.001
Lubrication	4.2 [3.3–4.8]	5.7 [5.1-6]	-12.5	0.63	< 0.001
Orgasm	2.8 [1.6-4]	5.2[4.8-6]	-13.61	0.69	< 0.001
Satisfaction	3.6 [2.4–4.8]	5.2[4.8-6]	-13.76	0.7	< 0.001
Pain	4.4 [2.8–5.6]	6 [4.8-6]	-9.21	0.47	< 0.001
Total FSFI-RO Score	21.9 [17.5–24.4]	30.8 [28.7–32.4]	-16.7	0.85	< 0.001

FSD: Female Sexual Dysfunction; FSFI-RO: Female Sexual Function Index Romanian Version; *Mann-Whitney U test

Regarding the factor structure of the Romanian FSFI questionnaire, a value of 0.926 was obtained in the Kaiser-Meyer-Olkin, with a statistically significant Bartlett sphericity test (p < 0.001).

To explore the factor structure, an exploratory factor analysis (EFA) was initially performed, leading to the identification of a six-factor structure. Subsequently, a confirmatory factor analysis (CFA) was conducted to validate the identified factor structure.

First, we used a Promax rotation with a minimum eigenvalue of 1.0 as a criterion factor extraction, and then four factors were identified with a minimum eigenvalue of 1.161 and a total of 75.83% of the variance: desire, arousal/satisfaction/pain, lubrication, and orgasm. A secondary principal component analysis was employed based on the studies of the original FSFI and the previous validation studies [55] that decided a six-factor structure.

The six-factor solution accounted for a total of 83.4% of the variance and the lowest eigenvalue of 0.537, included the domains: desire, arousal, lubrication, orgasm, satisfaction, and pain (Table 5). In this six-factor analysis, the minimum factor loading was 0.642. This six-factor model’s fit indices were in the acceptable range, indicating that the Romanian version of the FSFI can measure the same domains as the original questionnaire.

**Table 5:** Exploratory Factor Analysis of the FSFI-RO: factorial validity (n = 385)

	Factors					
	F1	F2	F3	F4	F5	F6
Q01 Desire: frequency	− 0.011	0.015	0.025	0.034	0.949	− 0.046
Q02 Desire: level	− 0.014	0.009	− 0.037	− 0.079	0.870	0.209
Q03 Arousal: frequency	0.065	0.063	− 0.004	0.098	0.039	0.715
Q04 Arousal: level	0.060	0.038	− 0.017	− 0.069	0.247	0.741
Q05 Arousal: confidence	− 0.068	− 0.091	0.050	0.230	0.042	0.735
Q06 Arousal: satisfaction	0.267	0.029	− 0.035	0.113	− 0.079	0.660
Q07 Lubrication: frequency	− 0.055	0.642	0.006	− 0.035	− 0.078	0.429
Q08 Lubrication: difficulty	− 0.025	0.880	− 0.007	0.120	0.112	− 0.134
Q09 Lubrication: frequency of maintaining	− 0.063	0.762	0.021	− 0.140	− 0.085	0.325
Q10 Lubrication: difficulty in maintaining	0.135	0.901	0.006	0.098	0.037	− 0.233
Q11 Orgasm: frequency	0.856	0.020	0.005	− 0.098	− 0.081	0.209
Q12 Orgasm: difficulty	0.936	0.037	− 0.009	− 0.069	0.038	− 0.093
Q13 Orgasm: satisfaction	0.843	− 0.052	0.015	0.169	0.016	− 0.006
Q14 Satisfaction: with closeness with partner	− 0.071	0.101	− 0.045	0.961	− 0.094	− 0.017
Q15 Satisfaction: with sexual relationship	0.019	0.019	0.015	0.776	− 0.021	0.190
Q16 Satisfaction: with overall sex life	0.069	− 0.072	0.031	0.756	0.121	0.107
Q17 Pain: frequency during vaginal penetration	0.045	0.041	0.929	− 0.019	0.041	− 0.045
Q18 Pain: frequency following vaginal penetration	− 0.019	0.001	0.945	− 0.012	0.009	0.008
Q19 Pain: level during or following vaginal penetration	− 0.021	− 0.024	0.916	0.013	− 0.055	0.045
Eigenvalue	9.665	2.079	1.503	1.161	0.901	0.537
% of explained variance	50.86	10.94	7.91	6.11	4.74	2.82

Principal Components Analysis using Promax rotation method with Kaiser normalization. Highest items’ factor loading for each component are shaded. F1: Orgasm, F2: Lubrication, F3: Pain, F4: Satisfaction, F5: Desire, F6: Arousal

Given that the exploratory factor analysis (EFA) showed that the six-factor model is a good fit, we continue to analyze the model by employing Confirmatory Factor Analysis (CFA). For the CFA we used goodness of fit indices to evaluate the model which showed a good fit (χ2(137, N = 385) = 347.231, p < 0.001). We got a Comparative Fit Index (CFI) value of 0.966, and a Tucker-Lewis Index (TLI) value of 0.957, which are good fit indices for the internal validity. The value of Standardized Root Mean Square Residual (SRMR) is 0.033, below the value of 0.08, which is generally considered a good fit [56]. All of the estimate coefficients loadings are significant and all variances have a positive sign which is good. This six-factor model’s fit indices are in the acceptable range, indicating that the Romanian version of the FSFI can measure the same domains as the original questionnaire.

### Internal consistency

To evaluate the internal consistency of the FSFI-RO, Cronbach’s α coefficients were determined for the total score and the domain scores, and were found to be high. In the domains, they ranged from 0.644 to 0.906. The coefficients for the domains and the total FSFI-RO score are presented in Table 6 along with 95%CI. Additionally, for the evaluation of internal consistency, we also used McDonald’s Omega which showed a solid internal consistency, ω = 0.943 with 95CI% [0.931; 0.952].

**Table 6:** Internal consistency for domains scores and total FSFI-RO score (n = 385)

	FSD group (n = 158)	Control group (n = 227)	Total Group (n = 385)
Desire	0.898 (0.847–0.919)	0.824 (0.767–0.867)	0.886 (0.868–0.913)
Arousal	0.821 (0.750–0.847)	0.644 (0.441–0.851)	0.904 (0.858–0.942)
Lubrication	0.847 (0.794–0.889)	0.729 (0.660–0.777)	0.890 (0.869–0.905)
Orgasm	0.854 (0.795–0.897)	0.844 (0.803–0.879)	0.921 (0.904–0.934)
Satisfaction	0.846 (0.778–0.895)	0.764 (0.679–0.827)	0.901 (0.869–0.925)
Pain	0.906 (0.889–0.921)	0.897 (0.861–0.911)	0.924 (0.909–0.936)
Total FSFI-RO Score	0.867 (0.825–0.903)	0.766 (0.705–0.815)	0.944 (0.932–0.954)

FSD: Female Sexual Dysfunction; FSFI-RO: Female Sexual Function Index Romanian Version; Cronbach’s α test. The round brackets present the 95%CI for α

### Reliability

In order to evaluate the test-retest reliability of the Romanian version, 50 women (19 from the FSD group and 31 from the control group) completed the Romanian version of the FSFI questionnaire twice within 4 weeks. The results presented in Table 7 showed high test-retest reliability in all cases, with ICC from 0.942 (95%CI 0.897;0.967) to 0.991 (95%CI 0.984;0.995) in the domains and 0.987 (95%CI 0.978;0.993) in the total score.

**Table 7:** Test-retest reliability for the domains and the total FSFI-RO score (n = 50)

	ICC	95%CI for ICC
Desire	0.942	0.897;0.967
Arousal	0.984	0.972;0.991
Lubrication	0.991	0.984;0.995
Orgasm	0.979	0.964;0.988
Satisfaction	0.983	0.970;0.990
Pain	0.986	0.976;0.992
Total FSFI-RO Score	0.987	0.978;0.993

ICC: interclass correlation coefficients; CI: confidence interval; FSFI-RO: Female Sexual Function Index Romanian Version

## Discussion

The field of sexuality in Romania is controversial due to numerous cultural, moral, and religious beliefs. Being a conservative, traditionalist society, the problems in this field are extremely rarely approached and studied, in most cases shame being the basis for neglecting this area. Moreover, the patriarchal society further accentuates this shame in the case of women. FSD has been shown to have a great impact on quality of life. Therefore, we considered that validating a psychometric tool for Romanian speaking population would be essential, facilitating women’s access to addressing sexual issues and widening openness on the subject.

In this study, we used similar methods to those used in other FSFI’s validity versions. We measured the reliability and validity of the Romanian version of the questionnaire by first translating the original version, and then evaluating its psychometric properties in a female sexually active sample.

The prevalence of FSD was found to be high in our sample, 41% of women meeting the DSM-5 criteria for sexual dysfunction at the time of the interview. Estimates of FSD prevalence vary widely across studies, but overall, it is recognized as a common condition that can significantly impact women’s quality of life. A comprehensive review published in 2016 analyzed the prevalence of FSD and reported an overall prevalence ranging from 20 to 60% [57].

The test-retest reliability proved to be high in all cases, in each domain as well as in the total score, showing excellent test-retest agreement. This suggests that the questionnaire consistently measures female sexual function over time in a reliable manner. The high test-retest reliability implies that if the same individuals were to complete the Romanian FSFI on two separate occasions, they would obtain similar scores, indicating stability in their reported sexual function. The internal consistency of the Romanian FSFI was also determined to be high. Internal consistency refers to the extent to which the items within a questionnaire consistently measure the same construct. In this case, the FSFI-RO demonstrated good internal consistency not only for the separate domains but also for the overall total score. This indicates that the items within each domain, as well as the questionnaire as a whole, are measuring the intended aspects of female sexual function consistently and reliably.

Convergent construct validity proved to be moderate to high in desire, arousal, lubrication, orgasm, and satisfaction domains, and weak in the pain domain. Convergent construct validity refers to the degree to which the FSFI domains correlate with other measures or indicators that assess similar constructs of sexual function. In the desire, arousal, lubrication, orgasm, and satisfaction domains, the FSFI-RO demonstrated moderate to high correlations with other established measures of sexual function, suggesting that these domains are capturing the intended aspects of female sexual function and are conceptually aligned with other validated measures [22, 24, 32, 33]. The positive correlations indicate that higher scores on the FSFI-RO domains are associated with better sexual function, providing evidence of convergent construct validity. However, in the pain domain, the FSFI-RO showed weaker correlations with other measures of pain or pain-related aspects of sexual function. This suggests that the pain domain of the FSFI-RO may not align as strongly with other established measures of pain or pain-related sexual dysfunction. Further investigation may be needed to explore the reasons for the weaker convergent validity in the pain domain and to identify potential improvements or modifications that could enhance its validity in assessing pain-related sexual issues.

Additionally, the inter-domain correlation values were lower than the correlations observed for the total score. This indicates that while there are moderate to high correlations within each domain, the relationships between different domains of the FSFI-RO are not as strong. This suggests that the domains of the FSFI-RO are relatively independent and are measuring distinct aspects of female sexual function. The lower inter-domain correlations further support the multidimensional nature of the questionnaire, with each domain representing a specific component of sexual function. These results can also be observed in the original study [16].

In the present study, analysis of discriminant construct validity has been evaluated by comparing the domains score and total score of the FSD group and the control group. Our results showed significantly higher scores in the control group, compared to the FSD group, in all domains, as well as in the total score. These findings indicate that the FSFI-RO successfully discriminates between women with sexual dysfunction and those without. The significantly higher scores in the control group suggest better sexual functioning in this group compared to the FSD group. The observed differences in scores provide evidence for the discriminant construct validity of the FSFI-RO, demonstrating its ability to accurately differentiate between individuals with and without sexual dysfunction. The results support the clinical utility of the FSFI-RO as a valid tool for identifying and distinguishing individuals with sexual difficulties from those without. By using the questionnaire, clinicians and researchers can effectively classify individuals into appropriate groups based on their sexual function scores, enabling targeted interventions, treatment planning, and monitoring of outcomes. This method has also been adopted in others validations, like original, the Spanish, the Chinese, the Malay, and Iranian [16, 24, 30, 33, 35], and, as in these studies, our results showed that the scores for each domain and full-scale of FSFI are significantly higher for the FSD group as compared to control group. The ability of the FSFI-RO to differentiate between clinical and nonclinical groups of women supports the high discriminant validity of the FSFI-RO.

Regarding the principal component analysis, at first, four factors were identified. In the literature, there is inconsistent factor solution in the validated FSFI versions, as factor quantity varies from 3 to 6 [25, 32, 34]. A secondary principal component analysis was employed, based on the studies of the original FSFI and the previous validation studies, that decided a six-factor structure [16, 24, 25, 30]. The first four factors identified, with a minimum eigenvalue of 1.161 and a total of 75.83% of the variance were: desire, arousal/satisfaction/pain, lubrication, and orgasm. These factors represent different aspects of female sexual function, and their inclusion aligns with previous studies on FSFI validation. The six-factor solution accounted for a total of 83.4% of the variance and the lowest eigenvalue of 0.537, included the domains: desire, arousal, lubrication, orgasm, satisfaction, and pain. The inclusion of these factors allows for a more comprehensive assessment of female sexual function, capturing a wider range of domains that are relevant to sexual experiences and satisfaction. The findings of the PCA in the present study contribute to the understanding of the factor structure of the FSFI-RO. The identified factors provide insights into the specific aspects of female sexual function that the questionnaire aims to measure. By employing a six-factor structure, the FSFI-RO accounts for a significant proportion of the variance, suggesting that these factors adequately represent the key dimensions of sexual function in the Romanian population.

Our research contributes to the field of female sexuality and sexual medicine in Romania, for research, as well as teaching purposes and in clinical practice. The clinical utility of the FSFI-RO lies in its potential to enhance the assessment and understanding of FSD in clinical practice and research settings. By providing a standardized and validated measurement tool to the Romanian population, the FSFI-RO can contribute to more accurate diagnosis, treatment planning, and evaluation of treatment outcomes in women experiencing sexual difficulties. In addition to diagnostic purposes, the Romanian FSFI can be used as an outcome measure in clinical trials or intervention studies. Its sensitivity to change allows researchers and clinicians to evaluate the effectiveness of various treatments or interventions for female sexual dysfunction. By administering the Romanian FSFI before and after treatment, researchers can assess the impact of interventions on different aspects of sexual function and quantify the magnitude of change. This not only contributes to the evidence base for treatment effectiveness but also helps guide clinical decision-making. Moreover, the availability of the Romanian FSFI can facilitate cross-cultural and international research collaborations. Researchers and clinicians in Romania can use the same standardized measurement tool as their counterparts in other countries, allowing for direct comparisons and meta-analyses across different cultural and linguistic contexts. This enables a more comprehensive understanding of the global prevalence, determinants, and consequences of female sexual dysfunction. Our study strongly supports the findings regarding the original English FSFI version.

The relatively high number of participants enrolled in the present study and the homogeneity of the study sample are the main strengths of this study. Some limitations of the study would be that we recruited our patients from an endocrinology clinic; therefore, the study group may not be representative of the Romanian female population. Sexual orientation of the participants was not assessed. Also, ethnic minority women were not excluded from this study. Therefore, we should be cautious about applying the FSFI-RO version for these women. We suggest that further cross-validation studies of the FSFI-RO version be conducted in women with different backgrounds and medical statuses. Another limitation of our study is the fact that constructs such as sexual well-being and sexual self-esteem were not measured and analyzed in relationship to our measure scores, even though previous studies have identified connections between these aspects and FSD. Such an analysis would have provided a more robust construct validity indication. Despite the limitations, our findings support the original study and previous FSFI validations and indicate that the Romanian version of the FSFI can measure the same domains as the original questionnaire.

## Conclusions

The FSFI-RO showed good psychometric properties, similar to those of the original English version. It is a reliable and valid instrument that can be used in Romanian-speaking women in order to assess FSD.

## Data Availability

The data presented in this study are available on request from the corresponding author. The data are not publicly available due to reasons concerning privacy of the subjects.

## Abbreviations

FSD: Female sexual dysfunction
FSFI: Female Sexual Function Index
FSFI-RO: Romanian Version of the Female Sexual Function Index
DSM-5: Diagnostic and Statistical Manual of Mental Disorders, 5th edition
ICC: Interclass correlation coefficients
KMO: Kaiser-Meyer-Olkin
CFA: Confirmatory factor analysis
CFI: Comparative fit index
TLI: Tucker-Lewis index
SRMR: Standardized Root Mean Square Residual
EFA: Exploratory factor analysis
CI: Confidence interval

## References

[1] McCool-Myers M, Theurich M, Zuelke A, Knuettel H, Apfelbacher C (2018). Predictors of female sexual dysfunction: a systematic review and qualitative analysis through gender inequality paradigms. BMC Womens Health.

[2] Brotto L, Atallah S, Johnson-Agbakwu C, Rosenbaum T, Abdo C, Byers ES, Graham C, Nobre P, Wylie K (2016). Psychological and interpersonal dimensions of sexual function and dysfunction. J Sex Med.

[3] McCool ME, Zuelke A, Theurich MA, Knuettel H, Ricci C, Apfelbacher C (2016). Prevalence of female sexual dysfunction among Premenopausal Women: a systematic review and Meta-analysis of Observational Studies. Sex Med Rev.

[4] Salonia A, Munarriz RM, Naspro R, Nappi RE, Briganti A, Chionna R, Federghini F, Mirone V, Rigatti P, Goldstein I, Montorsi F. Women’s sexual dysfunction: a pathophysiological review. BJU Int. 2004;93(8):1156-64. 10.1111/j.1464-410X.2004.04796.x. PMID: 15142131. 10.1111/j.1464-410X.2004.04796.x 15142131

[5] Edwards WM, Coleman E. Defining sexual health: a descriptive overview. Arch Sex Behav., Munarriz RM, Naspro R, Nappi RE, Briganti A, Chionna R, Federghini F, Mirone V, Rigatti P, Goldstein I, Montorsi F. Women’s sexual dysfunction: a pathophysiological review. BJU Int. 2004 May;93(8):1156-64. 10.1111/j.1464-410X.2004.04796.x. PMID: 15142131. 10.1111/j.1464-410X.2004.04796.x 15142131

[6] American Psychiatric Association (2013). Diagnostic and statistical Manual of Mental Disorders.

[7] Grover S, Shouan A (2020). Assessment Scales for sexual Disorders—A review. J Psychosexual Health.

[8] Derogatis LR. The Derogatis Interview for Sexual Functioning (DISF/DISF-SR): an introductory report. J Sex Marital Ther. 1997 Winter;23(4):291–304. doi: 10.1080/00926239708403933. PMID: 9427208. 10.1080/00926239708403933 9427208

[9] Taylor JF, Rosen RC, Leiblum SR (1994). Self-report assessment of female sexual function: psychometric evaluation of the brief index of sexual functioning for women. Arch Sex Behav.

[10] Rosen R, Brown C, Heiman J, Leiblum S, Meston C, Shabsigh R, Ferguson D, D’Agostino R Jr. The Female Sexual Function Index (FSFI): a multidimensional self-report instrument for the assessment of female sexual function. J Sex Marital Ther. 2000 Apr-Jun;26(2):191–208. doi: 10.1080/009262300278597. PMID: 10782451. 10.1080/009262300278597 10782451

[11] Isidori AM, Pozza C, Esposito K, Giugliano D, Morano S, Vignozzi L, Corona G, Lenzi A, Jannini EA (2010). Development and validation of a 6-item version of the female sexual function index (FSFI) as a diagnostic tool for female sexual dysfunction. J Sex Med.

[12] Clayton AH, McGarvey EL, Clavet GJ (1997). The changes in sexual functioning questionnaire (CSFQ): development, reliability, and validity. Psychopharmacol Bull.

[13] Thirlaway K, Fallowfield L, Cuzick J (1996). The sexual activity questionnaire: a measure of women’s sexual functioning. Qual Life Res.

[14] Forbes MK, Baillie AJ, Schniering CA (2014). Critical flaws in the female sexual function index and the international index of erectile function. J Sex Res.

[15] Esmat Hosseini S, Ilkhani M, Rohani C, Nikbakht Nasrabadi A, Ghanei Gheshlagh R, Moini A (2022). Prevalence of sexual dysfunction in women with cancer: a systematic review and meta-analysis. Int J Reprod Biomed.

[16] Mendonça CR, Arruda JT, Noll M, Campoli PMO, Amaral WND (2017). Sexual dysfunction in infertile women: a systematic review and meta-analysis. Eur J Obstet Gynecol Reprod Biol.

[17] Pérez-López FR, Ornat L, López-Baena MT, Pérez-Roncero GR, Tajada-Duaso MC, Chedrau P (2020). Association of female genital mutilation and female sexual dysfunction: a systematic review and meta-analysis. Eur J Obstet Gynecol Reprod Biol.

[18] Neijenhuijs KI, Hooghiemstra N, Holtmaat K, Aaronson NK, Groenvold M, Holzner B, Terwee CB, Cuijpers P, Verdonck-de Leeuw IM (2019). The female sexual function index (FSFI)-A systematic review of Measurement Properties. J Sex Med.

[19] Meston CM, Freihart BK, Handy AB, Kilimnik CD, Rosen RC (2020). Scoring and interpretation of the FSFI: what can be learned from 20 years of use?. J Sex Med.

[20] Wiegel M, Meston C, Rosen R. The female sexual function index (FSFI): cross-validation and development of clinical cutoff scores. J Sex Marital Ther. 2005 Jan-Feb;31(1):1–20. doi: 10.1080/00926230590475206. PMID: 15841702. 10.1080/00926230590475206 15841702

[21] Filocamo MT, Serati M, Li Marzi V, Costantini E, Milanesi M, Pietropaolo A, Polledro P, Gentile B, Maruccia S, Fornia S, Lauri I, Alei R, Arcangeli P, Sighinolfi MC, Manassero F, Andretta E, Palazzetti A, Bertelli E, Del Popolo G, Villari D (2014). The female sxual function index (FSFI): linguistic validation of the italian version. J Sex Med.

[22] Sánchez-Sánchez B, Navarro-Brazález B, Arranz-Martín B, Sánchez-Méndez Ó, de la Rosa-Díaz I, Torres-Lacomba M. The Female Sexual Function Index: Transculturally Adaptation and Psychometric Validation in Spanish Women. Int J Environ Res Public Health. 2020;17(3):994. doi: 10.3390/ijerph17030994. Erratum in: Int J Environ Res Public Health. 2020 Jun 17;17(12): PMID: 32033334; PMCID: PMC7037847. 10.3390/ijerph17030994 PMC7037847 32033334

[23] Wylomanski S, Bouquin R, Philippe HJ, Poulin Y, Hanf M, Dréno B, Rouzier R, Quéreux G (2014). Psychometric properties of the french female sexual function index (FSFI). Qual Life Res.

[24] Zachariou A, Filiponi M, Kirana PS (2017). Translation and validation of the greek version of the female sexual function index questionnaire. Int J Impot Res.

[25] Thiel Rdo R, Dambros M, Palma PC, Thiel M, Riccetto CL, Ramos Mde F. Tradução para português, adaptação cultural e validação do Female Sexual Function Index [Translation into Portuguese, cross-national adaptation and validation of the Female Sexual Function Index]. Rev Bras Ginecol Obstet. 2008;30(10):504 – 10. Portuguese. 10.1590/s0100-72032008001000005. PMID: 19082387. 10.1590/s0100-72032008001000005 19082387

[26] Hock M, Farkas N, Tiringer I, Gitta S, Németh Z, Farkas B (2019). Validation and translation of the hungarian version of the female sexual function index (FSFI-H). Int Urogynecol J.

[27] Nowosielski K, Wróbel B, Sioma-Markowska U, Poręba R. Development and validation of the Polish version of the Female Sexual Function Index in the Polish population of females. J Sex Med. 2013;10(2):386 – 95. 10.1111/jsm.12012. Epub 2012 Dec 4. PMID: 23211010. 10.1111/jsm.12012 23211010

[28] Sun X, Li C, Jin L, Fan Y, Wang D (2011). Development and validation of chinese version of female sexual function index in a chinese population-a pilot study. J Sex Med.

[29] Anis TH, Gheit SA, Saied HS, Al kherbash SA (2011). Arabic translation of female sexual function index and validation in an egyptian population. J Sex Med.

[30] Ghassamia M, Asghari A, Shaeiri MR, Safarinejad MR. Validation of psychometric properties of the Persian version of the female sexual function index. Urol J. 2013 Spring;10(2):878–85. PMID: 23801471. 23801471

[31] Sidi H, Abdullah N, Puteh SE, Midin M (2007). The female sexual function index (FSFI): validation of the malay version. J Sex Med.

[32] Takahashi M, Inokuchi T, Watanabe C, Saito T, Kai I (2011). The female sexual function index (FSFI): development of a japanese version. J Sex Med.

[33] Fakhri A, Pakpour AH, Burri A, Morshedi H, Zeidi IM (2012). The female sexual function index: translation and validation of an iranian version. J Sex Med.

[34] Ho TTT, Le MT, Truong QV, Nguyen VQH, Cao NT (2020). Validation of the vietnamese translation version of the female sexual function index in infertile patients. Sex Med.

[35] Rehman KU, Asif Mahmood M, Sheikh SS, Sultan T, Khan MA (2015). The female sexual function index (FSFI): translation, validation, and cross-cultural adaptation of an Urdu Version FSFI-U. Sex Med.

[36] Shahraki Z, Tanha FD, Ghajarzadeh M (2018). Depression, sexual dysfunction and sexual quality of life in women with infertility. BMC Womens Health.

[37] Pacik PT, Geletta S (2017). Vaginismus Treatment: clinical trials follow up 241 patients. Sex Med.

[38] Grzybowska ME, Wydra D. Responsiveness of two sexual function questionnaires: PISQ-IR and FSFI in women with pelvic floor disorders. Neurourol Urodyn. 2021;40(1):358–366. 10.1002/nau.24568. Epub 2020 Nov 4. PMID: 33150611. 10.1002/nau.24568 33150611

[39] Alsibiani SA, Rouzi AA (2010). Sexual function in women with female genital mutilation. Fertil Steril.

[40] Fuchs A, Czech I, Sikora J, Fuchs P, Lorek M, Skrzypulec-Plinta V, Drosdzol-Cop A (2019). Sexual functioning in pregnant women. Int J Environ Res Public Health.

[41] Kogure GS, Ribeiro VB, Lopes IP, Furtado CLM, Kodato S, Silva de Sá MF, Ferriani RA, Lara LADS, Maria Dos Reis R (2019). Body image and its relationships with sexual functioning, anxiety, and depression in women with polycystic ovary syndrome. J Affect Disord.

[42] Abu Ali RM, Al Hajeri RM, Khader YS, Shegem NS, Ajlouni KM. Sexual dysfunction in Jordanian diabetic women. Diabetes Care. 2008;31(8):1580-1. doi: 10.2337/dc08-0081. Epub 2008 May 5. PMID: 18458140; PMCID: PMC2494660. 10.2337/dc08-0081 PMC2494660 18458140

[43] Lunelli RP, Irigoyen MC, Goldmeier S. Hypertension as a risk factor for female sexual dysfunction: cross-sectional study. Rev Bras Enferm. 2018 Sep-Oct;71(5):2477–2482. 10.1590/0034-7167-2017-0259. PMID: 30304179. 10.1590/0034-7167-2017-0259 30304179

[44] Silva GMDD, Lima SMRR, Reis BFD, Macruz CF, Postigo S (2019). Evaluation of obesity influence in the sexual function of Postmenopausal Women: a cross-sectional study. Rev Bras Ginecol Obstet.

[45] Trompeter SE, Bettencourt R, Barrett-Connor E (2016). Metabolic syndrome and sexual function in Postmenopausal Women. Am J Med.

[46] Fuchs A, Matonóg A, Pilarska J, Sieradzka P, Szul M, Czuba B, Drosdzol-Cop A (2020). The impact of COVID-19 on female sexual health. Int J Environ Res Public Health.

[47] Bortun AC, Ivan V, Navolan DB, Dehelean L, Borlea A, Stoian D (2021). Thyroid autoimmune disease-impact on sexual function in Young Women. J Clin Med.

[48] Gherbon A, Frandes M, Roman D, Anastasiu-Popov D, Timar R (2020). Risk factors for sexual dysfunction in romanian women with type 1 diabetes mellitus and chronic autoimmune thyroiditis: a comparative cross-sectional study. Diabetol Metab Syndr.

[49] Laumann EO, Nicolosi A, Glasser DB, Paik A, Gingell C, Moreira E, Wang T, GSSAB Investigators’ Group. ;. Sexual problems among women and men aged 40–80 y: prevalence and correlates identified in the Global Study of Sexual Attitudes and Behaviors. Int J Impot Res. 2005 Jan-Feb;17(1):39–57. 10.1038/sj.ijir.3901250. PMID: 15215881. 10.1038/sj.ijir.3901250 15215881

[50] Allahdadi KJ, Tostes RC, Webb RC (2009). Female sexual dysfunction: therapeutic options and experimental challenges. Cardiovasc Hematol Agents Med Chem.

[51] Mokkink LB, Terwee CB, Patrick DL, Alonso J, Stratford PW, Knol DL, Bouter LM, de Vet HC. The COSMIN study reached international consensus on taxonomy, terminology, and definitions of measurement properties for health-related patient-reported outcomes. J Clin Epidemiol. 2010;63(7):737 – 45. doi: 10.1016/j.jclinepi.2010.02.006. PMID: 20494804. 10.1016/j.jclinepi.2010.02.006 20494804

[52] Cronbach LJ (1951). Coefficient alpha and the internal structure of tests. Psychometrika.

[53] Stephane C, Claus E, Peter D, Helios DR. Basic Functions for Power Analysis - Package ‘Pwr’ for R 2020, v1.3-0.

[54] de Winter JC, Dodou D, Wieringa PA. Exploratory Factor Analysis With Small Sample Sizes. Multivariate Behav Res. 2009 Mar-Apr;44(2):147 – 81. 10.1080/00273170902794206. PMID: 26754265. 10.1080/00273170902794206 26754265

[55] Opperman EA, Benson LE, Milhausen RR (2013). Confirmatory factor analysis of the female sexual function index. J Sex Res.

[56] Hu L, Bentler PM (1999). Cutoff criteria for fit indexes in covariance structure analysis: conventional criteria versus new alternatives. Struct Equation Modeling: Multidisciplinary J.

[57] Nappi RE, Cucinella L, Martella S, Rossi M, Tiranini L, Martini E (2016). Female sexual dysfunction (FSD): prevalence and impact on quality of life (QoL). Maturitas.

